# Induction of enhanced stem-directed neutralizing antibodies by HA2-16 ferritin nanoparticles with H3 influenza virus boost†

**DOI:** 10.1039/d4na00964a

**Published:** 2025-02-11

**Authors:** Qingyu Wang, Jiaojiao Nie, Zejinxuan Liu, Yaotian Chang, Yangang Wei, Xin Yao, Lulu Sun, Xiaoxi Liu, Qicheng Liu, Xinyu Liang, Xinran Zhang, Yong Zhang, Weiheng Su, Qi Zhao, Yaming Shan, Yingwu Wang, Xianbin Cheng, Yuhua Shi

**Affiliations:** a National Engineering Laboratory for AIDS Vaccine, School of Life Sciences, Jilin University Changchun Jilin 130012 China; b MTM Biotechnology Ltd Zhongshan Guangdong 528437 China; c High School Attached to Northeast Normal University Changchun Jilin 130012 China; d Faculty of Health Sciences, University of Macau Taipa Macau China; e MoE Frontiers Science Center for Precision Oncology, University of Macau Taipa Macau SAR China; f Key Laboratory for Molecular Enzymology and Engineering, The Ministry of Education, School of Life Sciences, Jilin University Changchun Jilin 130012 China; g Department of Thyroid Surgery, The Second Hospital of Jilin University Changchun China

## Abstract

Current seasonal influenza vaccines offer limited protection against influenza viruses due to genetic drift. The urgent need for a universal influenza vaccine to combat highly mutated strains is evident. This study utilized the conserved HA2 subunit of hemagglutinin (HA) and a short linear epitope of HA2 (HA2-16) from the H3 influenza virus to conjugate with ferritin, resulting in the construction of recombinant immunogens termed HA2-F and HA2-16-F, respectively. *In vitro* characterization confirmed the self-assembly of prokaryotically expressed HA2-F and HA2-16-F into nanoparticles (NPs). To simulate natural virus infection in the vaccinated population, intranasal infection with the whole H3N2 virus was administered as a final boost. Enhanced binding activity to A/Hong Kong/4801/2014 (H3N2) and A/17/California/2009/38 (H1N1) virus was detected in the HA2-16 group induced by the A/Wisconsin/67/2005 (H3N2) virus boost (Titer >10^4^). Furthermore, higher titers of neutralizing antibodies were elicited by HA2-16-F NP (ID_50_: 50.4–631.0) compared to those by HA2-F NP (ID_50_: 20.3–178.2). These results demonstrated that the H3N2 virus boost focused the antibody response on the HA2-16 epitope. Additionally, our immunization strategy was found to reduce serum ferritin reactive antibodies. In summary, HA2-16 not only holds promise as a vaccine candidate but also exhibits significant potential for influenza vaccine production, particularly in enhancing the levels of induced stem-directed antibodies. This study contributes to the development of recombinant immunogens for improved influenza vaccine efficacy.

## Abstract 2

HA2-16 ferritin nanoparticles induce stem-directed neutralizing antibodies by H3 influenza virus boost.

## Graphical abstract

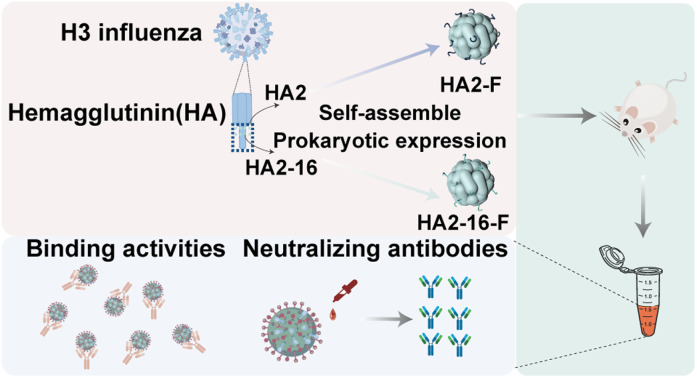

## Introduction

Nanotechnology has been demonstrated to enhance the protective efficacy of vaccines, including those for influenza, SARS-CoV-2, and human immunodeficiency virus (HIV).^1^ Recent innovations have demonstrated that several natural proteins possess the ability to form nanoparticles (NPs), making them ideal for antigen presentation and immunostimulation.^2–5^ Ferritin proteins, well-characterized protein cages, serve as a natural nanomaterial and have been extensively studied.^6,7^ Examples of drugs and agents that can be loaded onto ferritin include chemotherapeutic agents, genes, fluorescent molecules, and a variety of peptides that have been displayed on its surface.^8^ Other studies have demonstrated that ferritin, with its excellent biosafety profile, serves as an ideal scaffold for presenting trimeric antigens.^8,9^ Furthermore, the threefold symmetry axis of ferritin allows for antigen display in a nature-like conformation, a unique feature distinguishing ferritin from other particles.^10^ In the context of vaccine delivery, DNA vaccines have garnered widespread attention. The production of DNA vaccines is a straightforward method for generating large quantities of vaccine candidates, and these vaccines can effectively induce both humoral and cellular immune responses. Additionally, self-assembling ferritin NPs exhibit remarkable thermal and chemical stability,^11^ as well as high biocompatibility.^12^ The size of the NPs is a critical factor in nanomaterial synthesis and their applications in nanotechnology. For example, superparamagnetic iron oxide nanoparticles (SPION) with sizes ranging from 10 to 20 nm demonstrate effective biological activity.^13,14^ Recent research has focused on utilizing ferritin as a vehicle for drug and vaccine delivery systems, as well as exploring its potential as a novel structure-based material.^15^

The influenza virus is a significant respiratory pathogen capable of evading the immune system, leading to substantial global morbidity and mortality.^16,17^ The most effective countermeasure to prevent influenza virus infections is vaccination, aiming to induce a robust antibody response against the viral hemagglutinin (HA) glycoprotein. HA is an important trimeric glycoprotein on the surface of the influenza virus and consists of HA1 and HA2, which could fold into the globular head domain and stem domain. HA1 is highly mutable, while HA2 is more conserved. Seasonal influenza vaccines could primarily elicit immune responses against the immunodominant region HA1, resulting in a narrow breadth of protection.^18–21^ To enhance the breadth of protection, the development of next-generation vaccines has focused on targeting highly conserved viral structures, particularly the conserved HA. Concurrently, advanced strategies are needed to optimize these conserved antigens to improve their immunogenicity and protective efficacy.^22,23^

The mRNA vaccine platform, known for its rapid preparation and exceptional flexibility, not only allows for more time to gather data prior to strain selection but also eliminates issues related to egg-based production and host adaptation.^24^ A recent study A recent study demonstrated that a seasonal quadrivalent influenza mRNA-1010 vaccine encoding four hemagglutinin proteins induced higher hemagglutinin inhibition antibody titres against influenza A strains compared to a standard inactivated seasonal influenza vaccine.^25^ However, due to the high cost of mRNA vaccines, developers have been exploring low-cost, high-efficiency strategies for vaccine production.

In one approach, a full-length H1N1 influenza HA was genetically fused at the interface of adjacent ferritin subunits, enabling spontaneous assembly into an HA-NP with a mean diameter of 37.23 nm.^26^ This strategy offers a promising platform for vaccine development. Building on this, we have re-engineered ferritin-based NPs to improve their assembly efficiency and optimize their ability to elicit a broad and potent immune response.

This strategy offers a promising platform for vaccine development. Building on this, we have re-engineered ferritin-based NPs to improve their assembly efficiency and optimize their ability to elicit a broad and potent immune response,^27^ demonstrate that the stable H1 HA2 protein, when expressed in *E. coli*, can protect mice from lethal viral challenges.^28,29^ Additionally, previous research has shown that the conserved linear epitope of HA2 from the H3 influenza virus can induce potent neutralizing activity against the A/Kansas/04/2009 (H3N2) strain.^30^ In summary, HA-based vaccines, including HA trimer vaccines, HA2 vaccines, and epitope-based vaccines, represent promising candidates that could elicit broad and strong protection against influenza.

In this study, we designed a recombinant ferritin that incorporates a linear conserved stem epitope (HA2-16) derived from the HA2 region. The self-assembly properties of the recombinant proteins were characterized *in vitro*. We then assessed the immunogenicity of the resulting NPs *in vivo*. Furthermore, the specific immunoenhancement was performed by a boost immunization with virus to evaluate immunogenicity and protective efficacy, which could decrease the production of non-neutralizing antibodies. We hope that this study will contribute to the development of efficient epitopes for universal influenza vaccines.

## Experimental

### Cells, viruses, and animals

Madin Darby canine kidney (MDCK) cells were adherently grown in Dulbecco's modified Eagle's medium (DMEM) with 10% fetal bovine serum (FBS) at 37 °C in 5% CO_2_. *E. coli* Trans 5α and BL21 (DE3) competent cells were purchased from TRANSGEN Biotech (Beijing, China). A/17/California/2009/38 (H1N1), A/Wisconsin/67/2005 (H3N2), B/56/Brisbane/60/08 were obtained from Changchun BCHT Pharmaceutical Co., Ltd. HA2-16-KLH (HA2-16 coupled with keyhole limpet hemocyanin protein) was preserved in our laboratory. Female BALB/c mice (18–20 g) were purchased from Liaoning Changsheng Biotechnology Co., Ltd (Liaoning, China). All animals were specific pathogen-free and maintained in the experimental animal facility at Jilin University. The animal experiments were conducted according to the regulations provided by the Animal Care and Use Committee of Jilin University (Ethical number: YNPZSY2023096).

### Plasmid construction of ferritin-based recombinant proteins

All non-identical, full-length influenza sequences (a total of 2000 sequences for H3N2) were obtained from the NCBI Influenza Virus Database. The sequences were aligned using MEGA (version 11). The aligned sequences were then imported into Weblogo (https://weblogo.berkeley.edu/logo.cgi, version 2.8.2) to generate sequence logos, which visually represent residue conservation at each position. Residue conservation a was further analyzed using the ConSurf web server (https://consurf.tau.ac.il/) and mapped onto the crystal structure of H3N2 (PDB ID code 2YPG) using PyMOL (version 2). The target gene from A/Kansas/04/2009 (H3N2) was fused to the N-terminus of ferritin, with a 6× His-tag added to the C-terminus of ferritin for affinity purification. The gene encoding a fusion protein consisting of HA2 and ferritin (HA2-F) was optimized and synthesized into a pET-20b (+) vector by GENSCRIPT Biotech (Nanjing, China) (Fig. S5†). The plasmids were constructed using polymerase chain reaction (PCR), with *Nde I* and *Xho I* serving as the restriction sites for cloning.

### Production of ferritin-based recombinant proteins

The production and purification scheme of the NPs is summarized in Fig. S1.† The above recombinant plasmids were transformed into *E. coli* BL21 competent cells for protein expression. The positive strains were selected and cultured in 4 mL of Luria–Bertani (LB) medium containing 50 μg mL^−1^ ampicillin at 37 °C overnight. Subsequently, the overnight enriched bacterial solution was transferred to 1 L fresh LB medium containing 50 μg mL^−1^ ampicillin until the OD 600 of the culture reached 0.6 to 0.8. Then, 1 mM isopropyl-β-D-thiogalactoside (IPTG; Takara, China) was added to induce the expression of protein, and the cells were incubated overnight at 25 °C. The harvested bacterial cultures were centrifuged and ultrasonically disintegrated at 4 °C.

### Preparation and characterization of NPs

The supernatant was filtered through a 0.22 μm syringe filter and then purified using an affinity His-Trap™ column (GE Healthcare, USA). The purified proteins self-assembled into NPs *via* stepwise dialysis and were stored at −80 °C. Sodium dodecyl sulfate–polyacrylamide gel electrophoresis (SDS-PAGE) (12% polyacrylamide gel) was performed to assess the NPs, followed by Coomassie blue staining (Sigma-Aldrich, USA) and western blotting with an anti-His tag antibody (TRANSGEN Biotech HT501-01, Beijing, China) to confirm the identity of the purified proteins. The samples were visualized using the BeyoECL Moon kit (Beyotime Biotechnology, China) and imaged with a Tanon-5200 Chemiluminescent Imaging System (Tanon Science and Technology, Shanghai, China).

The morphology of NPs was characterized by transmission electron microscopy (TEM) (H-7650, Hitachi, Japan). Briefly, the samples were loaded on 200-mesh carbon-coated copper grids and negatively stained with 0.5% phosphotungstic acid. TEM imaging was performed at acceleration voltages of 80 kV and 100 kV, and images (at 40 000× magnification) were captured using a charge-coupled device (CCD) camera system. The particle size distribution of NPs in phosphate-buffered saline (PBS) was determined by dynamic light scattering (DLS) using a Zetasizer NANO ZS90 instrument (Malvern, Worcestershire, UK). The concentrated sample underwent further purification through size exclusion chromatography (SEC) utilizing an ÄKTA pure system equipped with a Superdex 200 increase 10/300 GL (GE Healthcare, USA). The chromatography was conducted in 150 mM NaCl, pH 7.5 at a flow rate of 0.3 mL min^−1^. Dextran blue (Sigma-Aldrich, USA) and thyroglobulin (Master of Bioactive Molecules, USA) were used as the molecular weight standards.

### Endotoxin testing

The mGEL method was used to determine endotoxin contamination (Zhanjiang A&C Biological Ltd). The purified protein was diluted to a concentration of 1 mg mL^−1^. Standard solutions and the purified protein were then added to the mGEL assay. After incubation for 1 hour, the samples were observed for any solidification. The results indicated that the endotoxin level was below 5 EU mL^−1^.

### Animal immunization and virus boost

Female BALB/c mice were randomly divided into four groups, with six mice in each group: mock, ferritin NPs (negative control), and ferritin-based recombinant NPs. Prior to each immunization, 20 μg of each NP formulation was diluted in 100 μL of PBS and mixed with an equal volume of squalene-based oil-in-water emulsions, resembling the composition of the MF59 adjuvant (Novartis). MF59 contains 2.5 mL of Tween 80, 2.5 mL of Span 85, 21.5 mL of squalene, and 473.5 mL of sodium citrate. Serum samples were collected every two weeks, and the mice were euthanized at week 10. Mice were immunized subcutaneously (s.c.) four times at 2 weeks intervals. Two weeks after the fourth immunization, under pentobarbital sodium anesthesia, the H3N2 virus (A/Wisconsin/67/2005) was introduced intranasally as a booster. Mice were monitored daily for weight changes over a 10 days period. Two weeks after the boost, serum samples were collected for analysis.

Lung tissues were harvested 4 days post-boost, and viral titers as well as hematoxylin and eosin (H&E) staining were evaluated. The tissues were homogenized in cold PBS using a homogenizer, and viral titers were determined by TCID_50_ assay in MDCK cells. The collected lung tissues were then fixed in 4% paraformaldehyde, embedded in paraffin, and sectioned. The tissue sections were stained with H&E and examined under an ECLIPSE Ci-L plus microscope (Nikon, Japan).

### Mouse serological analysis by enzyme-linked immunosorbent assay (ELISA)

A total of 100 TCID_50_ of A/17/California/2009/38 (H1N1), A/Wisconsin/67/2005 (H3N2), and B/56/Brisbane/60/08 viruses (Changchun BCHT Pharmaceutical Co., Ltd), along with 0.2 μg per well of H3N2-A/Brisbane/10/2007 and H3N2-A/Wyoming/03/2003 HA proteins (Sino Biological, Inc., Beijing, China), were coated onto 96-well plates. The plates were washed with PBST (0.05% Tween-20 in PBS) and blocked with 3% bovine serum albumin (BSA) in PBS at 37 °C for 2 h. Then, the plates were washed twice with PBST. Then, the plates were washed twice with PBST.

Next, serial dilutions of mouse serum in PBS were added to the 96-well plates and incubated at 37 °C for 1 hour. After four washes with PBST, horseradish peroxidase (HRP)-conjugated goat anti-mouse IgG (Dingguo Inc., Beijing, China) in 1% BSA-PBS was added to the plates and incubated at 37 °C for 45 minutes. Following another four washes with PBST, 100 μL per well of 3,3′,5,5′-tetramethylbenzidine (TMB) color development solution (TRANSGEN Biotech, Beijing, China) was added, and the plates were incubated for 20 minutes at room temperature in the dark. The reaction was stopped with 2 M H_2_SO_4_, and the absorbance at 450 nm was measured using an iMarK™ microplate reader (Bio-Rad).

### Neutralizing activity detection of immunogen

Neutralizing activity was assessed using microneutralization (MN) assays. The assay was performed as previously described with modifications.^31,32^ Briefly, MDCK cells were seeded at 10^5^ cells per well in a 96-well plate. Two-fold serial dilutions of mouse serum samples were prepared in a virus culture medium. The diluted serum samples were then mixed with 100 TCID_50_ of A/Wisconsin/67/2005 (H3N2), A/Hong Kong/4801/2014 (H3N2), A/17/California/2009/38 (H1N1) or B/56/Brisbane/60/08 (B) viruses and incubated at 37 °C for 1 h.

The mixture of virus and serum was added to the MDCK cell monolayer in the 96-well plate and incubated at 37 °C for 48 hours to observe the cytopathic effect (CPE). The neutralizing antibody titer was calculated as the median inhibitory dose (ID_50_) using the Reed and Muench method.

### Detection of hemagglutination inhibition (HAI) titer

The HAI titer was determined using an HAI assay, performed as previously described with modifications.^33^ Serum samples were first treated with receptor destroying enzyme (RDE; Denka Seiken) at 37 °C for 20 h and then heat-inactivated at 56 °C for 30 min. Next, 4 HA units of A/Wisconsin/67/2005 or A/Hong Kong/4801/2014 virus were mixed with serial dilutions of serum and incubated at 37 °C for 30 min.

After incubation, 50 μL of 1.5% chicken red blood cells in sterile PBS was added to each well, and the mixture was incubated for an additional 30 minutes at 37 °C. The HAI titer was defined as the reciprocal of the highest serum dilution at which no agglutination was observed.

### Statistical analysis

Statistical analyses were performed using GraphPad Prism 8.0 (GraphPad Software, San Diego, CA, USA). The results are expressed as the mean ± standard deviation (SD). Ordinary one-way ANOVA was used to compare differences between groups. Statistical significance was determined by Tukey's multiple comparisons test unless otherwise stated (indicated as follows: *P < 0.05; **P < 0.01; ***P < 0.001; ns, not statistically significant).

## Results

### Characterization of recombinant proteins and nanoparticles

**Fig. 1:**
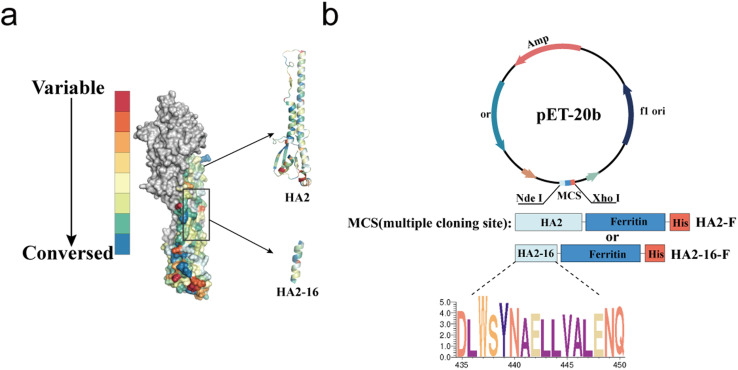
Conservation analysis and plasmid construction of immunogens. (a) Conservation rates of H3N2 HA sequences isolated from 2000 to 2021. Sequences are highlighted in a colored crystal structure model of HA (PDB ID: 2YPG). (b) Plasmid construction of the prokaryotic expression systems of pET-20b-HA2-F and pET-20b-HA2-16-F.

**Fig. 2:**
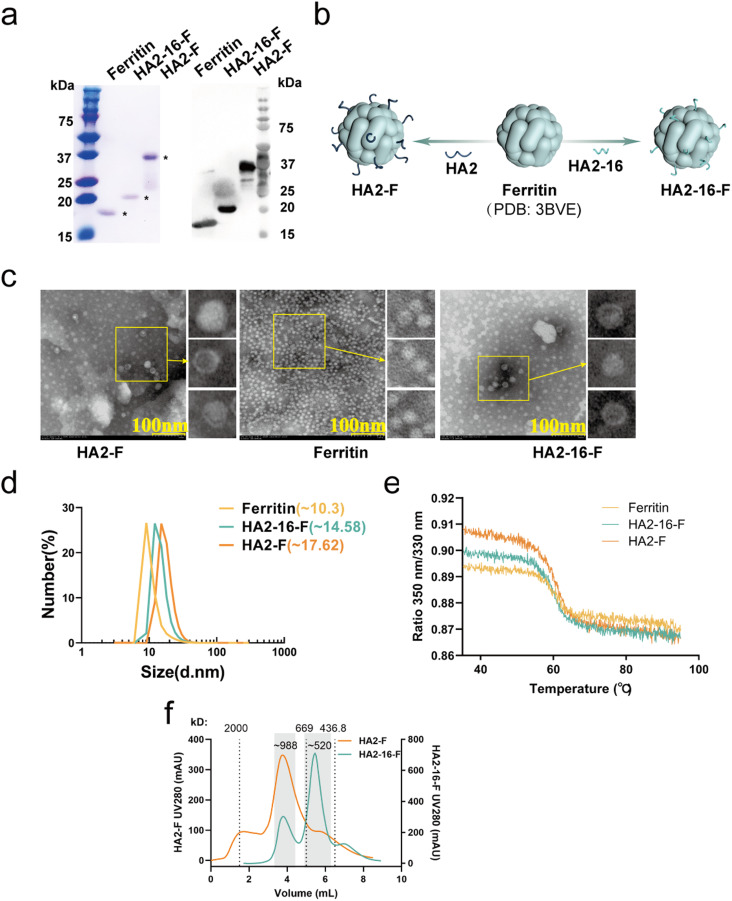
Characterization of self-assembled NPs. (a) SDS-PAGE and Coomassie blue staining of ferritin (∼18.2 kDa), HA2-16-F (∼21.7 kDa) and HA2-F (∼41.2 kDa). (b) 3D illustration of the structure of three self-assembled recombinant ferritin, HA2-F and HA2-16-F. (c) Morphological analysis of HA2-F, ferritin, and HA2-16-F by TEM. (d) Size analysis of ferritin, HA2-16-F, and HA2-F by DLS. (e) Unfolding profiles of ferritin, HA2-F and HA2-16-F. (f) SEC analysis of HA2-F and HA2-16-F nanoparticles was performed using dextran blue (2000 kDa) and thyroglobulin (669 kDa) as the molecular weight standards. Analysis of the elution profiles (indicated region) yielded calculated molecular weights of ∼988 kDa for HA2-F and ∼520 kDa for HA2-16-F nanoparticles.

As previously reported, highly conserved targets (HA2-16) from the H3N2 virus were identified through sequence alignment and conservation analysis (Fig. 1a).^34^ To investigate whether trimerization could enhance the immunogenicity of HA2-16, we constructed genes encoding the conserved antigens, which were fused to the N-terminus of *Helicobacter pylori* ferritin, followed by a 6× His-Tag at the C-terminus of ferritin (HA2-F and HA2-16-F) (Fig. 1b). SDS-PAGE analysis of recombinant proteins showed the expected monomeric molecular weight of 41.2 kDa (HA2-F) and 21.7 kDa (HA2-16-F) (Fig. 2a and S2†).

Based on the 3D structures of the nanoparticles (NPs) (Fig. 2b), TEM images showed that both HA2-F and HA2-16-F monomers were capable of self-assembling into a dispersive, sphere-like morphology, similar to the ferritin (Fig. 2c). The DLS results revealed a single, narrow distribution of HA2-F and HA2-16-F cages, indicating that the particles were uniform in size (Fig. 2d). Furthermore, the unfolding profiles of the NPs were measured, showing similar unfolding curves, which suggests that the NPs have stable and comparable structures (Fig. 2e). Notably, freshly purified NPs and those stored at −80 °C for 6 months exhibited nearly identical unfolding profiles, indicating that the NPs remained stable after long-term storage (Fig. S1†). SEC and native PAGE analysis supported the presence of aggregated nanoparticles in the sample, confirming the formation of HA-F nanoparticles (Fig. 2f, S3 and S4†). HA-NPs also had the expected apparent molecular weight of (HA2-F: ∼988 kDa; HA2-16-F: ∼520 kDa) consistent with a ferritin-based nanoparticle displaying eight copies of the HA2 monomers. These findings demonstrated that we have successfully constructed and expressed self-assembling HA2-F and HA2-16-F NPs in *E. coli*.

### Enhanced antigen-specific immune responses elicited by H3N2 virus boost in mice

**Fig. 3:**
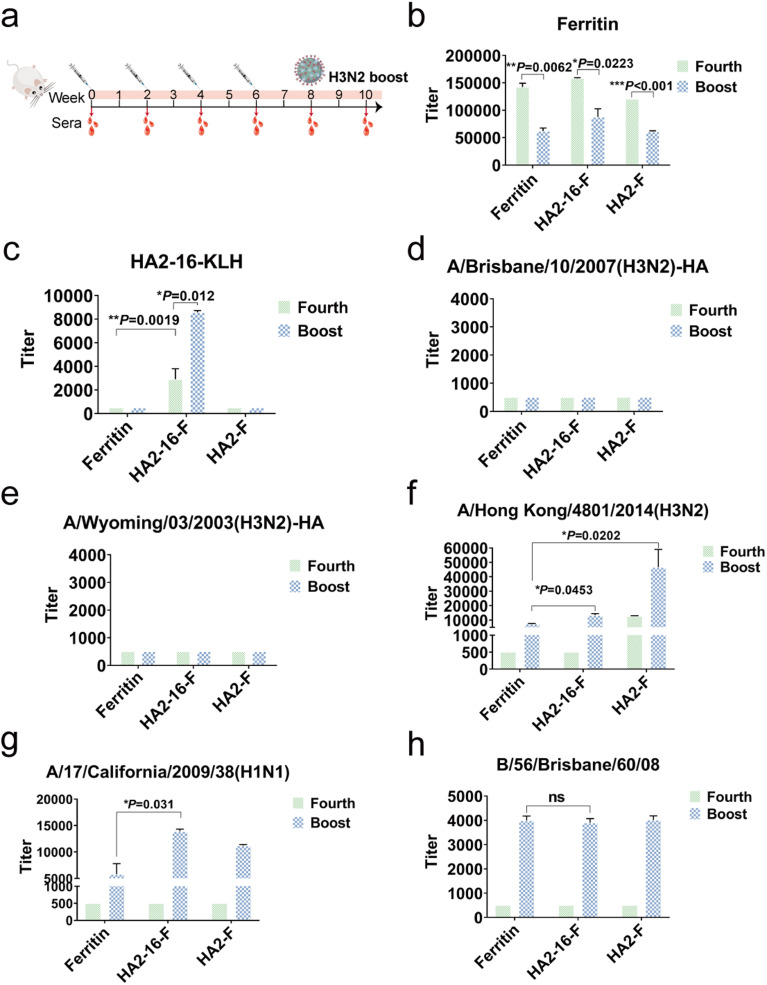
Humoral immune responses in mice. (a) Mouse immunization and blood collection schedule. (b and c) Binding activities of mouse sera with purified ferritin and HA2-16-KLH proteins. (d and e) Binding activities of mouse sera with H3N2-A/Brisbane/10/2007 and H3N2-A/Wyoming/03/2003 HA proteins. (f–h) Binding activities of mouse sera with A/Hong Kong/4801/2014, A/17/California/2009/38 and B/66/Brisbane/60/08 virus. One-way ANOVA then Tukey's multiple comparison tests were utilized for statistical significance analysis (indicated as follows: *P < 0.05; **P < 0.01; ***P < 0.001; ns, not statistically significant).

To evaluate the immunogenicity of NP vaccination, BALB/c mice were subcutaneously immunized four times at two-week intervals with a 20 μg dose of NPs formulated in MF59 adjuvant. Mice vaccinated with equal volumes of MF59 adjuvant alone were used as the mock group. Serum samples were collected two weeks after each immunization. To simulate natural viral infection in the vaccinated population, the whole H3N2 virus was administered intranasally as the final boost (Fig. 3a). We quantified serum HA antibody levels using ELISAs coated with full-length HA proteins, serum ferritin-reactive antibodies using ELISAs coated with purified ferritin proteins, and serum HA2-16-reactive antibodies using ELISAs coated with HA2-16-KLH.

Interestingly, H3N2 virus infection significantly reduced serum ferritin-reactive antibodies, which may mitigate the impact of anti-ferritin antibodies on the immunogenicity of the NPs (Fig. 3b). As expected, the HA2-16-specific antibodies remained at similar levels following the H3N2 boost (Fig. 3c), but no antibodies recognizing full-length HA proteins were produced (Fig. 3d and e). However, the HA2-F group exhibited higher levels of HA-specific antibodies against the A/Hong Kong/4801/2014 (H3N2) virus compared to the HA2-16-F group. Additionally, binding activity to both A/Hong Kong/4801/2014 (H3N2) and A/California/17/2009 (H1N1) viruses was detected in the HA2-16 group induced by the H3N2 boost (Fig. 3f and g), but no binding to B/56/Brisbane/60/08 (B) virus was observed (Fig. 3h).

To investigate whether the viral booster could redirect immune responses toward immunodominant epitopes on the HA head domains, serum HA head-specific antibodies were measured using hemagglutination inhibition (HAI) assays. The results showed that no significant HAI titers were detected (Table S1†).

Taken together, these results demonstrate that animals in the HA2-16 group, infected with the H3N2 virus, were able to generate antibodies reactive to both H3N2 and H1N1 viruses.

### Enhanced broadly neutralizing antisera and protective efficacy induced by HA2-16 NP

**Fig. 4:**
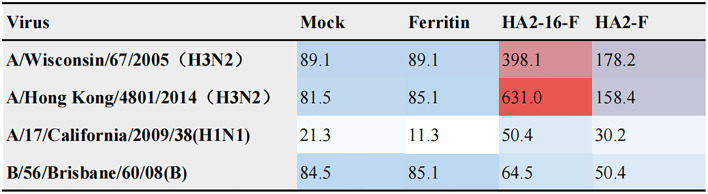
Neutralizing activity (ID_50_) against different viruses. Virus neutralization potency of sera was evaluated in a standard neutralization assay against A/Wisconsin/67/2005 (H3N2), A/Hong Kong/4801/2014 (H3N2), A/17/California/2009/38 (H1N1) or B/56/Brisbane/60/08 (B) influenza viruses.

Neutralizing antibodies inhibit viral invasion by binding tightly to viral structures, thereby providing potent immune protection.^35^ As shown in Fig. 4, sera from HA2-16-F-immunized mice bound more strongly to both homologous and heterologous HA than sera from HA2-F-immunized mice. These results demonstrate that HA2-16-F NP effectively induces neutralizing antibodies, rather than non-neutralizing antibodies.

**Fig. 5:**
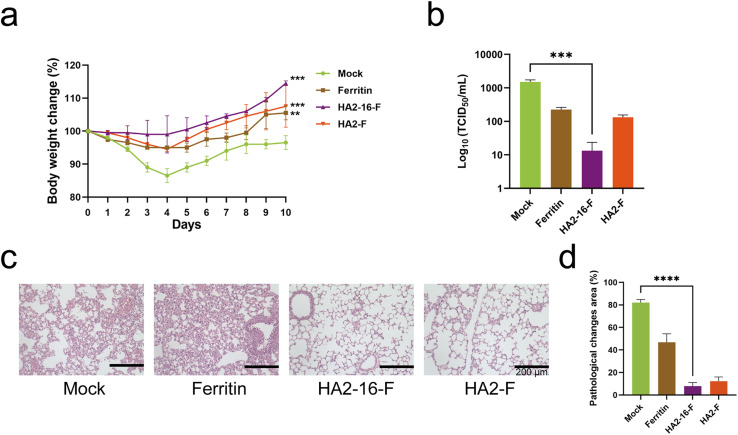
Protective immunity of NPs. (a) Body weight change of mice immunized post H3N2 boost, *n* = 6. (b) The lung viral titers were determined by a TCID_50_ assay on day 4 after the challenge (*n* = 3). (c) Pathological analysis on day 4 after the boost (*n* = 3). The lung changes in MF59-adjuvanted NPs (ferritin, HA2-16, and HA2-F) and the BALB/c mice administered with MF59-adjuvanted PBS (mock). Scale bar = 200 μm. (d) The quantitative analysis of inflammatory cells changes area.

Body weight measurements revealed no significant weight loss in any of the groups (Fig. 5a). Lung pathology analysis showed no significant pathological damage in the MF59-adjuvanted HA NP groups. In contrast, the mock group (PBS mixed with MF59) and the MF59-adjuvanted ferritin groups exhibited severe cell infiltration and alveolar wall congestion (Fig. 5b). Additionally, significantly lower viral loads in the lung tissues were observed in HA2-16-F-immunized mice compared to the mock group (Fig. 5c and d).

## Discussions

Broadly protective NP vaccines are urgently needed to address the high variability of the main antigenic determinants of the influenza virus. The highly conserved HA stem domain represents a promising target for universal vaccines, offering better protection against pandemic strains.^36^ Previous studies have demonstrated the successful development of the HA2-16 epitope-based NP vaccine, which enhances neutralizing activity.^30,34,37^ Research has also shown that the ferritin platform can display exogenous peptides and self-assemble into nanoparticles.^38–42^ The introduction of ferritin stabilizes and enhances the immunogenicity of the HA epitope. However, the presence of anti-ferritin antibodies may interfere with the immunogenicity of the NP. Interestingly, we found that boosting with the whole virus could reduce anti-ferritin antibodies and mitigate the issue of anti-vector immunity.

Studies have show that “original antigenic sin” can suppress the immune response to vaccines.^43–45^ We propose that immunization with HA stem NP, followed by a boost with the whole virus, could simulate natural viral infection. Our results demonstrate that boosting with the H3N2 virus shifts the antibody response toward the HA2-16 epitope, potentially reducing the production of non-neutralizing antibodies and minimizing the risk of antibody-dependent enhancement (ADE). Moreover, the strategy of removing immunodominant epitopes could elicit broadly neutralizing responses directed at conserved, yet subdominant, epitopes.^46^

Our proposed immunization strategy could effectively boost low levels of pre-existing stalk-directed antibodies. A similar humoral immune response may also be elicited in humans with an appropriate vaccination protocol, taking into account the binding affinity of different human HLA alleles. However, there are limitations to our animal model, and four rounds of immunization followed by a boost may not be acceptable for human use. We plan to further evaluate the potential adverse reactions to our immunization strategy and optimize it for future human applications. For example, additional studies are needed to improve the efficiency of the antibody response or to design a more conserved immunogen.

Finally, NP vaccines themselves have demonstrated adjuvant properties. The conserved antigenic surfaces of NP vaccines mimic the pathogen structures to which the host immune system has evolved to respond.^47^ We also proved that ferritin could ensure the binding between our epitopes and the stem region of HA. The removal of immune dominant epitopes could further promote the production of neutralizing antibody. It will be important to verify the immune response to NP vaccines in the absence of additional adjuvants.

Based on the induction of high-titer antibodies against the stem fragment, our findings strongly suggest that the HA2-16 epitope could be used in a vaccine candidate designed to boost low levels of pre-existing stem-directed antibodies. Considering the immunogenicity of the displayed antigen will help further expand the application potential of ferritin nanoparticle platforms.

## Conclusions

In conclusion, ferritin-based recombinant proteins were successfully expressed in prokaryotic organisms, without the mammalian glycosylation modifications or other post-translational modifications typically seen in viral proteins. Both the HA2 domain and the HA2-16 epitope, when coupled with ferritin, were able to self-assemble into NPs *in vitro*. HA2-16 ferritin nanoparticles induced enhanced stem-directed neutralizing antibodies, which were further boosted by the whole virus. The H3N2 virus boost effectively focused the antibody response on the HA2-16 epitope, potentially reducing the production of non-neutralizing antibodies and overcoming the issue of anti-vector immunity. These findings suggest that HA2-16 has the potential to be developed into a broad-spectrum influenza vaccine candidate.

## Data availability

The data supporting this study are available within the article and ESI.†

## Author contributions

QYW, JJN, XBC and YHS conceptualized the study. JJN, YTC and YWW developed the methodology. YGW, XY, LLS, XXL, YHS, QCL, XYL and XRZ performed the investigation. QYW wrote the article. YWW and YMS edited the manuscript. WHS, QZ and YZ supervised the study. YHS, YMS and WHS acquired funds. YZ provided MF59 adjuvant. All authors approved the final version of the manuscript. The manuscript was written through contributions of all authors.

## Conflicts of interest

There are no conflicts to declare.

## Supplementary Material

NA-OLF-D4NA00964A-s001
