# Supplementary material for: Induction of enhanced stem-directed neutralizing antibodies by HA2-16 ferritin nanoparticles with H3 influenza virus boost
Source: Nanoscale Adv. 2025 Feb 11;7(7):2011–20. doi: 10.1039/d4na00964a (PMC11833233; doi:10.1039/d4na00964a)
Supplement: NA-007-D4NA00964A-s001 [file NA-007-D4NA00964A-s001.pdf]

Supplementary Information (SI) for Nanoscale Advances.
This journal is © The Royal Society of Chemistry 2025

Supporting Information

Induction of Enhanced Stem-directed Neutralizing Antibodies by HA2-16 Ferritin nanoparticles with H3 Influenza Virus Boost

Qingyu Wang [a], *Jiaojiao Nie* [a], *Zejinxuan Liu* [a], *Yaotian Chang* [a], *Yangang Wei* [b], *Xin Yao* [a], *Lulu Sun* [a], *Xiaoxi Liu* [a], *Qicheng Liu* [c], *Xinyu Liang* [c], *Xinran Zhang* [c], *Yong Zhang* [a], *Weiheng Su* [a], *Qi Zhao* [d, e], *Yaming Shan* [a, f], *Yingwu Wang* [f], *Xianbin Cheng* [g, *], *Yuhua Shi* [a, *]

[a] National Engineering Laboratory for AIDS Vaccine, School of Life Sciences, Jilin University, Changchun, Jilin 130012, China.

[b] Microthome Biotechnology Ltd., Zhongshan, Guangdong 528437, China

[c] High school attached to northeast normal university, Changchun, Jilin 130012, China.

[d] Faculty of Health Sciences, University of Macau, Taipa, Macau, China.

[e] MoE Frontiers Science Center for Precision Oncology, University of Macau, Taipa, Macau SAR, China.

[f] Key Laboratory for Molecular Enzymology and Engineering, The Ministry of Education, School of Life Sciences, Jilin University, Changchun, Jilin 130012, China.

[g] Department of Thyroid Surgery, The Second Hospital of Jilin University, Changchun, China

*Correspondence

X. B. Cheng, Department of Thyroid Surgery, The Second Hospital of Jilin University, Changchun, China

E-mail: chengxb19@mails.jlu.edu.cn

Y. H. Shi, School of Life Sciences, Jilin University, No. 2699 Qianjin Street, Changchun, Jilin 130012, China

E-mail: yhshi@jlu.edu.cn

KEYWORDS. H3 influenza virus, Hemagglutinin, Short linear epitope, Self-assembling, Nanoparticle, Ferritin

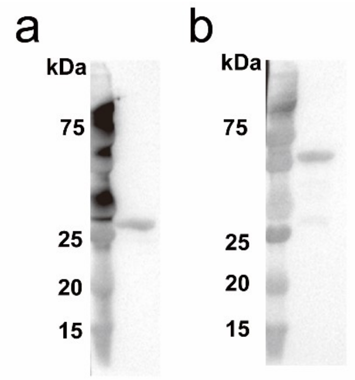

Figure S1. Determination of the integrity and stability of nanoparticles. Western blot analysis of (a) HA2-16-F using anti-HA2-16 peptide detection antibody and (b) HA2-F using anti-H3 influenza virus detection antibody.

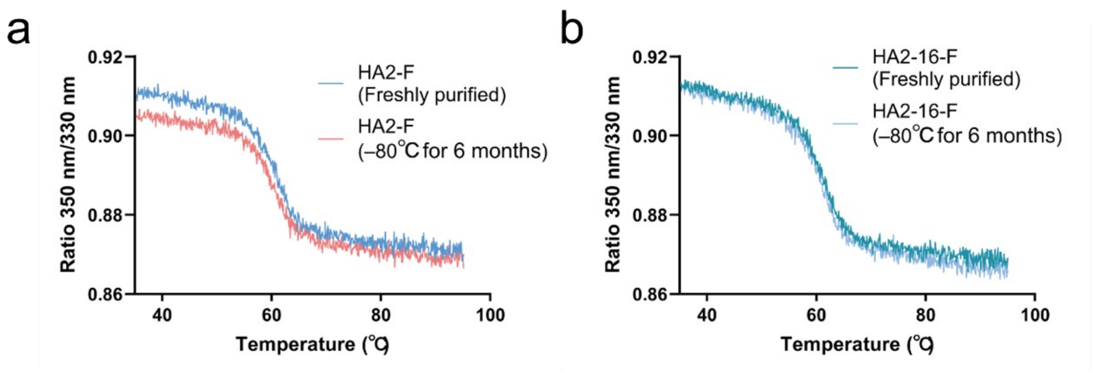

Figure S2. Unfolding profiles of freshly purified proteins and the proteins stored at -80°C for 6 months. (a) HA2-F, (b) HA2-16-F.

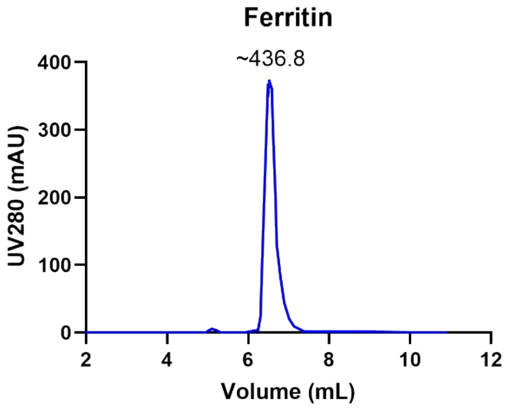

Figure S3. SEC analysis of ferritin nanoparticles. Calculated molecular weight using retention volumes of the ferritin nanoparticles was shown (~436.8 kDa).

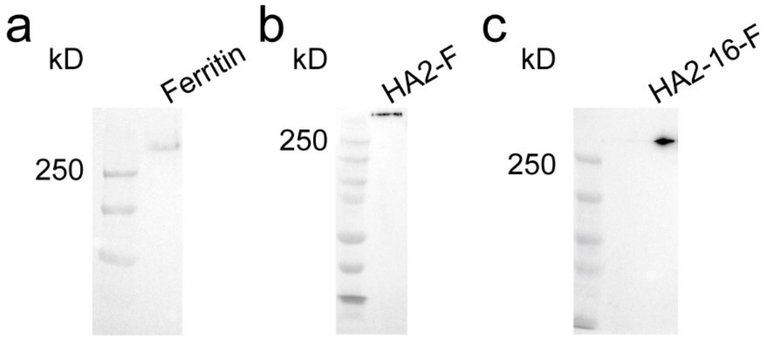

Figure S4. 8% Native-PAGE and western-blotting of purified NPs. (a) Native-PAGE of purified Ferritin NPs. (b) Native-PAGE of purified HA2-F NPs. (c) Native-PAGE of purified HA2-16-F NPs.

pET-20b-HA2-Ferritin

GIFGAIAGFIENGWEGMVDGWYGFRHQNSEGRGQAADLKSTQAAIDQINGKLNRLIGKTNEKFHQIEKEFS
EVEGRIQDLEKYVEDTKIDLWSYNAELLVALENQHTIDLTDSEMNKLFEKTKKQLRENAEDMGNGCFKIYHK
CDNACIGSIRNGTYDHDVYRDEALNNRFQIKEFGSGDIIKLLNEQVNKEMNSSNLYMSMSSWCYTHSLDGA
GLFLFDHAAEEYEHAKKLIIFLNENNVPVQLTSISAPEHKFEGLTQIFQKAYEHEQHISESINNIVDHAIKSKDH
ATFNFLQWYVAEQHEEEVLFKDILDKIELIGNENHGLYLADQYVKGIAKSRKSGGGGSGGGGSGGGGSHH
HHHH

pET-20b-HA2-16-Ferritin

MDLWSYNAELLVALENQDIIKLLNEQVNKEMQSSNLYMSMSSWCYTHSLDGAGLFLFDHAAEEYEHAKKLII
FLNENNVPVQLTSISAPEHKFEGLTQIFQKAYEHEQHISESINNIVDHAIKSKDHATFNFLQWYVAEQHEEEVL
FKDILDKIELIGNENHGLYLADQYVKGIAKSRKSGGGGSGGGGSGGGGSHHHHHH

pET-20b-Ferritin

DIIKLLNEQVNKEMQSSNLYMSMSSWCYTHSLDGAGLFLFDHAAEEYEHAKKLIIFLNENNVPVQLTSISAPE
HKFEGLTQIFQKAYEHEQHISESINNIVDHAIKSKDHATFNFLQWYVAEQHEEEVLFKDILDKIELIGNENHGLY
LADQYVKGIAKSRKSGGGGSGGGGSGGGGSHHHHHH

Figure S5. The amino acid sequences of pET-20b-HA2-Ferritin, pET-20b-HA2-16-Ferritin, and pET-20b-Ferritin, the linker information was marked in yellow.

TABLE

Table S1. Serum HAI activity in the MF59 adjuvant immunization group

H3N2	Pre-immune	Mock	Ferritin	HA2-16-F	HA2-F
**A/Wisconsin/67/2005**	2^3	2^4	2^4	2^4	2^4
**A/Hong Kong/4801/2014**	2^3	2^4	2^4	2^4	2^4
